# Synergistic Strategies of Heat and Peroxyacetic Acid Disinfection Treatments for *Salmonella* Control

**DOI:** 10.3390/pathogens12111336

**Published:** 2023-11-09

**Authors:** Olja Šovljanski, Aleksandra Ranitović, Ana Tomić, Nenad Ćetković, Ana Miljković, Anja Saveljić, Dragoljub Cvetković

**Affiliations:** 1 Faculty of Technology Novi Sad, University of Novi Sad, Bulevar cara Lazara 1, 21000 Novi Sad, Serbia; a.ranitovic@uns.ac.rs (A.R.); anav@uns.ac.rs (A.T.); anja.saveljic@uns.ac.rs (A.S.); cveled@uns.ac.rs (D.C.); 2 Faculty of Medicine, University of Novi Sad, Hajduk Veljkova 3, 21000 Novi Sad, Serbia; nenad.cetkovic@mf.uns.ac.rs (N.Ć.); ana.miljkovic@mf.uns.ac.rs (A.M.)

**Keywords:** antimicrobial treatment, heat influence, peroxyacetic acid influence, Box–Behnken design, *Salmonella*

## Abstract

The food industry has recognized a pressing need for highly effective disinfection protocols to decrease the risk of pathogen emergence and proliferation in food products. The integration of antimicrobial treatments in food production has occurred as a potential strategy to attain food items of superior quality with respect to microbiological safety and sensory attributes. This study aims to investigate the individual and synergistic effects of heat and peroxyacetic acid on the inactivation of bacterial cells, considering various contact times and environmental conditions. Four *Salmonella* serotypes, isolated from industrial meat production surfaces, were employed as model organisms. By systematically assessing the impacts of individual factors and synergistic outcomes, the effectiveness of bacterial cell inactivation and the efficiency of heat and peroxyacetic acid could be predicted. To better approximate real-world food processing conditions, this study also incorporated a bovine albumin-rich condition as a simulation of the presence of organic loads in processing steps. The findings revealed the essential need for a synergistic interplay of investigated parameters with the following optimized values: 1.5% concentration of peroxyacetic acid, temperature range of 60–65 °C, and contact time of 3 min for the complete effect regardless of the degree of contamination.

## 1. Introduction

The food supply chain, encompassing growth, harvest, transportation, storage, and food preparation, often lacks adequate hygiene controls, making it a route of infection for both humans and animals [1]. Foodborne illnesses, stemming from contaminated food, present significant challenges in food production and supply systems. Pathogenic microorganisms that can be transmitted between humans and animals result in acute illnesses and economic losses [2]. Microbial contamination in food remains a global health threat, affecting a substantial percentage of individuals. A fundamental prerequisite for minimizing microbial contamination in food systems is the ongoing development of effective procedures for disinfection at the industrial level [3,4]. However, many microorganisms can survive chemicals and processing conditions used in the food industry, making appropriate regulations and hygiene training essential [5,6]. Many industrial procedures for disinfection often involve the use of growth-suppressing agents, but their long-term use leads to resistance, creating a much larger and longer-term problem in the food cycle for humans and animals [7]. This also led to the ineffectiveness of even specific antimicrobial agents against foodborne pathogens, while actual sources of new antimicrobial substances are very limited. [8,9,10]. In the past decades, synergistic strategies in disinfection protocols and new control measures have been investigated via a combination of many chemicals and physical parameters related to the food industry [11,12].

*Salmonella*, a significant food-related pathogen, poses a considerable economic burden. Its presence in food results from various sources, primarily through contaminated animal products and water, but sources of infection can also be humans and pets [13,14,15,16,17,18,19]. The frequency of *Salmonella* infection in animal farms and the primary production process depends primarily on the production system and the control measures applied [20,21,22]. Cross-contamination in animal products is a common source of salmonellosis. Numerous studies have identified slaughterhouses as potential sources of *Salmonella* cross-contamination between meat and industrial surfaces, equipment, utensils, and water, signifying a potential critical stage for controlling its transmission throughout the food chain [23,24,25,26,27,28]. In industrial settings, the continual processing of animals and meats and minimal hygienic steps in slaughterhouses can be the main reason for cross-contamination within the same flock or with previously slaughtered flocks [29]. Understanding these potential sources of contamination is pivotal for implementing effective control measures in the poultry industry. Primary methods for controlling *Salmonella* spp. at the farm level have relied on biosafety measures. Success in this effort hinges on thorough cleaning and disinfection, which involve the use of effective detergents, the thorough removal of organic remains, proper disinfectant concentrations, and high-quality cleaning water. Ensuring the strict and continuous sanitation of drinking water throughout the production cycle, implementing vaccination strategies, and utilizing symbiotics and acids in animal nutrition have also been essential [30]. On the other hand, the mentioned methods have not been able to prevent the spread of *Salmonella* spp. in animals and their derived products, which opened an essential need to develop and implement novel, cost- and time-effective, and efficient strategies for a combination of typical and new chemicals and operation parameters during cleaning and disinfection processes [30].

Synergistic strategies in cleaning and disinfection procedures are precisely what led to deflection on the individual efficacies of some chemical substances or operating parameters in mitigating *Salmonella*’s presence in diverse settings in the food industry. Moreover, the industrial application of the individual effect of growth-suppressing agents has presented certain challenges, particularly concerning the resilient nature of *Salmonella* in response to heat exposure, which is an often-used tool for the inactivation of microbial cells. This pathogen has demonstrated an unwavering resistance to heat, often demanding impractically long treatment durations, particularly at mild temperatures [31]. One more challenge related to heat influence on *Salmonella* spp. is highly dependent on the microorganism culture and matrix [32], which indicates potential inefficiency and discontinuity in the applied treatment. Concurrently, extensive research within the existing literature has showcased the remarkable effectiveness of peroxyacetic acid, even at low concentrations, in significantly reducing *Salmonella* contamination [33,34]. Due to the elevation of organic content during peroxyacetic acid decomposition, the presence of a pungent odor, and limited production and higher costs compared with conventional disinfection agents [35], the adequate and optimized use of this growth-suppressing agent is required.

This study seeks to answer the possibility of a combined disinfection approach that could offer a more practical, efficient, and industry-applicable solution to control *Salmonella* contamination. Recognizing that a single-pronged reliance on heat or peroxyacetic acid for *Salmonella* inactivation might not be a feasible or sustainable strategy, the purpose of this investigation is to carry out a comprehensive Box–Behnken evaluation of the synergistic effect of heat and peroxyacetic acid on four *Salmonella* serotypes for the first time. This robust strategy in binding two growth-suppressing agents presents an easy approach for defining not only the *Salmonella* serotype’s impact but also the influence of the proteinaceous interferer that is often present in industrial conditions. 

## 2. Materials and Methods

### 2.1. Chemicals and Consumables

All chemicals used in this study were commercially available products from ThermoFisher Scientific (Voltham, MA, USA) and HiMedia (Mumbai, India). All plastic consumables (Petri plates, plates, slides, etc.) were procured as sterile single-use materials. Contaminated materials were sterilized by moist heat in an autoclave and disposed of in accordance with good laboratory practice.

### 2.2. Devices

The necessary devices for performing the tests are as follows: centrifuge (Gyrozen 1580R, Gyrozen, Gimpo, Republic of Korea); water bath (GFL Gesellschaft Fuer Labortec, Meckenheim, Germany); incubators (GFL Gesellschaft Fuer Labortec, Meckenheim, Germany); homogenizer–vortex (GFL Gesellschaft Fuer Labortec, Meckenheim, Germany); DensiChek Plus nephelometer (BIOMERIEUX, Marcy-l’Étoile, France). After preparation, the necessary substrates and diluents were sterilized by moist heat in an autoclave (MASC, EET Ermafa Environmental Technologies GmbH, Vienna, Austria), while the glass material was subjected to dry sterilization in a desiccator (BioBase, Karnataka, India).

### 2.3. Bacterial Strains

Four different *Salmonella* serotypes were selected for this study. As presented in Table 1, these bacteria were previously isolated from working surfaces in the industrial conditions of primary pork production. Briefly, the strains were isolated in the 2020–2021 period using the ISO method “Microbiology of the food chain-Horizontal method for detection, number determination and serotyping of *Salmonella*-Part 1: Detection of *Salmonella* spp. (ISO 6579-1:2017)”. The serotyping of the selected isolates was performed via commercial analysis in accordance with the standard “Horizontal method for detection, counting, and typing of *Salmonella*-Part 3: Instructions for typing *Salmonella* spp.” (ISO/TR 6579-3:2014). The locations of isolation were slaughterhouse departments of a meat processing facility in the Republic of Serbia.

### 2.4. Preparation of Bacterial Suspension

Bacterial cultures, previously stored at refrigerator temperature, were refreshed on XLD (xylose lysine deoxycholate) agar for 24 h at 37 °C. A single colony from the XLD agar was transferred to 10 mL of TSB (tryptone soya broth) broth, followed by incubation at 37 °C. The concentration of the overnight culture was assessed using McFarland standards (targeted value was ~8 log CFU/mL), and the whole volume of the suspension was centrifuged at 8000 rpm for 8 min. Overnight incubation enables the obtainment of cells in a stationary phase. The mentioned physiological state is characterized by increased resistance to stress compared with cells in the exponential phase. The resulting pellet was washed many times with a sterile physiological solution, and it was used to prepare a suspension of the target concentration of bacterial cells (~7 log CFU/mL) in a test tube with a physiological solution. All prepared test suspensions were maintained at a temperature of 4 °C in the water bath and used within 2 h.

### 2.5. The Concept of Testing Biocidal Effects

**Figure 1:**
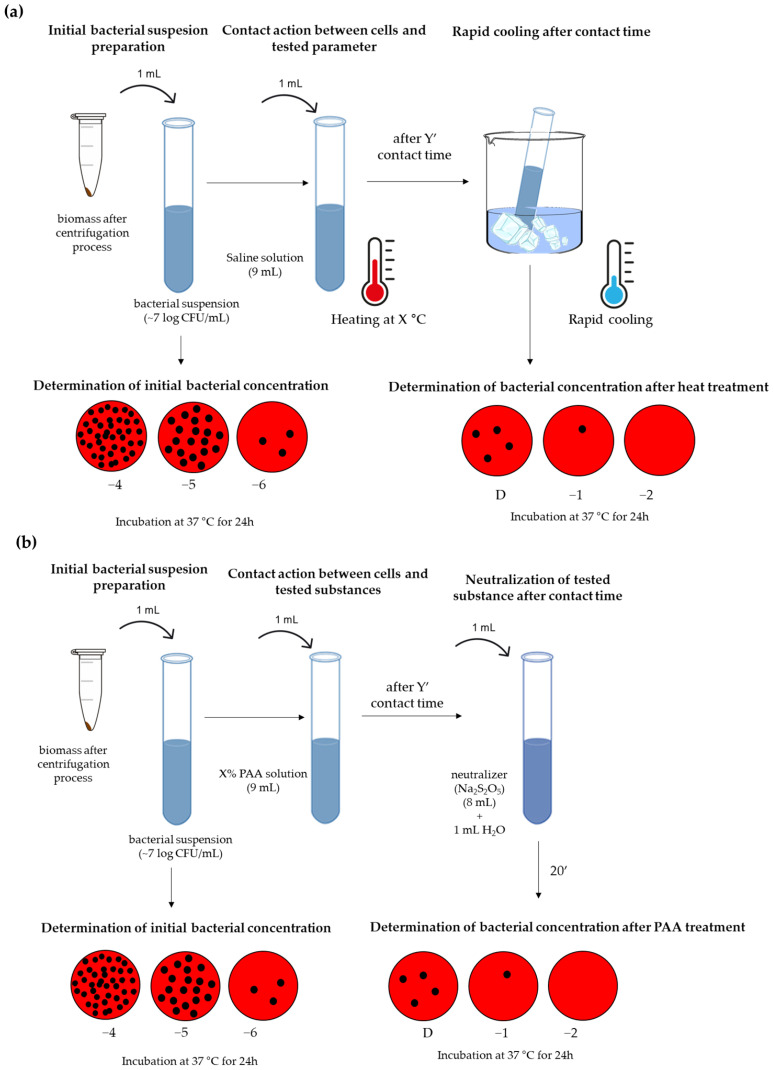
Examination of the biocidal effect: (**a**) heat; (**b**) peroxyacetic (PAA) acid.

The research concept (Figure 1) included three steps, and it intends to define the best combination of synergistic heat and peroxyacetic acid treatments and consequently establish an effective disinfection procedure. Contact time was defined as an additional variable during the test, and the results were processed in the form of kinetic models.

- step

This step included the screening of the effect of heat or peroxyacetic acid to assess the approximate limits of these parameters in which the biocidal effect occurs (Figure 1). The testing of the individual influence of heat (Figure 1a) includes testing common water temperature ranges used in the sanitation process. More precisely, temperatures of 45, 55, and 65 °C were tested, with a contact time of 1, 3, 5, and 10 min. The heat resistance of microorganisms can be affected by the method of heating, which is why it is possible to apply different techniques in evaluating the heat resistance of bacteria in liquid diluents [36,37]. These methods include heating in water baths using capillary tubes, test tubes, or glass ampoules that are partially or completely immersed in water; heating via a pasteurization process; heating apparatus with submerged coils, etc. [38]. In this research study, the test tube method was used, and the test tube was partially immersed in a heated water bath. The method was chosen because of its ease of handling and the ability to easily control the contact temperature. The cell suspension was exposed to direct contact with heated water in the bath. Also, the tubes with the diluent were pre-tempered at the target temperature so that there would be no temperature differences during the primary contact between hot water and the reaction tube. Bacterial concentrations at zero minute presented initial concentration values without heat influence.

When choosing the contact time, we took into account that there is a rapid and sudden cooling of the water after contact between hot water and working surfaces with temperatures that are much lower than the initial temperature of the water. The influence of peroxyacetic acid at room temperature included the testing of 1, 2, and 5% PAA solutions with contact times of 1, 3, 5, and 10 min (Figure 1b). According to the recommendation of the manufacturer of the PAA solution, sodium metabisulfite (Na_2_S_2_O_5_) was used for neutralization in the following ratio: 1.25 mg/L of Na_2_S_2_O_5_ should be added to 1 mg/L of PAA. Bacterial concentrations at zero minute presented initial concentration values without PAA influence.

II. step

To maximize the benefits of a synergistic approach, it is crucial to define and optimize various factors, not only tested growth-suppressing agent values but also a contact time. Based on the efficiency of the individual effect of heat and the concentration of peroxyacetic acid, an experimental plan was formed that included the following variables and levels: concentration of PAA solution (1, 1.5, and 2%), water temperature (heat) (55, 60, and 65 °C), and contact time (1, 3, and 5 min). In order to perform a comprehensive investigation, a Box–Behnken experimental design was used (Table 2).

III. step

**Figure 2:**
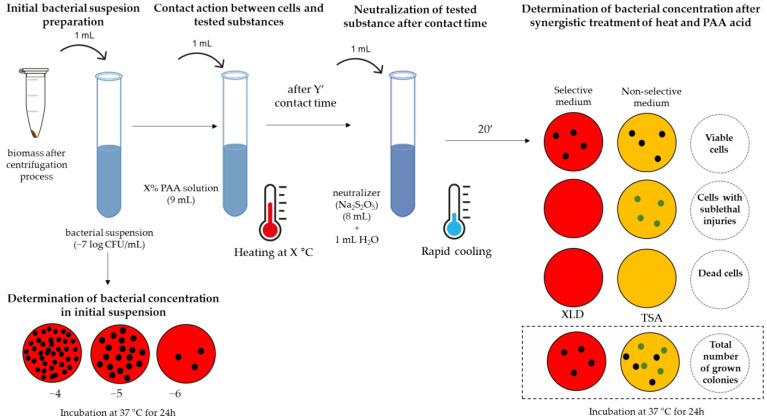
Scheme of testing the combined treatment of heat and peroxyacetic (PAA) acid.

The main purpose of testing antimicrobial treatments in laboratory conditions is to simulate the conditions of the use of the disinfection procedure as closely as possible. When a disinfectant manufacturer determines the use concentration for their product, it is necessary to recognize at least three factors that can affect the performance of the disinfectant in industrial conditions: contact time, temperature, and organic load [39]. The selected contact times and temperatures are adjusted to reflect the time that the disinfectant can remain on surfaces before runoff (and non-horizontal surfaces) at average room temperatures. However, the presence of organic matter on surfaces can reduce the activity of the disinfectant either via a chemical reaction with it or by blocking the physical access of the active component to the microbiological target [39]. Therefore, this step included an identical scenario as the previous step shown in Figure 2, but the influence of the so-called “dirty” conditions is included. This includes the interfering contamination of the reaction mixture with a bovine albumin solution in order to mimic the conditions of proteinaceous impurities. After preparing the test suspension, the albumin solution (3 g/100 mL) was mixed with an equivalent volume of the test suspension (1 mL + 1 mL) during a contact time of 2 min, followed by a contact step with the PAA solution (8 mL), and all further steps are shown in Figure 2. The used concentration of the interfering substance is recommended in reference [40]. As the ultimate goal of this step, a “worst case” scenario is set. Specifically, in the animal food processing industry, an average number of total bacteria greater than 6 log CFU/cm^2^ is observed before the cleaning process [37], while *Salmonella* detection occurs in 10 out of 18 surface swabs [41]. Also, an increased number of samples positive for the presence of *S. Typhimurium* by as much as 78% was observed in the presence of substances with a protein nature [42].

### 2.6. Mathematical Analysis

For the evaluation of the individual effect of heat or PAA concentrations, kinetic modeling was performed. The kinetics of bacterial concentrations during contact time with the tested killing agent was considered as a four-parameter sigmoidal mathematical model (Equation (1)) that is highly suitable for microbiological systems [43,44,45,46].

(1) $$y(t)=d+\frac{a-d}{1+{\left(\frac{t}{c}\right)}^{b}}$$

In Equation (1), the bacterial concentration (log CFU/mL) is represented as *y(t)*, whereas regression coefficients are denoted as follows: *a*—minimum of the experimentally obtained values (at *t* = 0); *d*—the maximally acquired value; *c*—the inflection point (the point between *a* and *d*); *b*—the Hill’s slope (the steepness of the inflection point (*c*)). Numerical verification was defined by the reduced chi-square statistic (χ^2^), root mean square error (RMSE), mean bias error (MBE), and mean percentage error (MPE). These parameters were calculated using Equations (2)–(5):

(2) $$\chi^{2}=\frac{\sum_{i=1}^{N}{\left(x_{exp,i}-x_{pre,i}\right)}^{2}}{N-n},$$

(3) $$RMSE={\left[\frac{1}{N}\cdot \sum_{i=1}^{N}{\left(x_{pre,i}-x_{exp,i}\right)}^{2}\right]}^{1/2},$$

(4) $$MBE=\frac{1}{N}\cdot \sum_{i=1}^{N}\left(x_{pre,i}-x_{exp,i}\right),$$

(5) $$MPE=\frac{100}{N}\cdot \sum_{i=1}^{N}\left(\frac{\left|x_{pre,i}-x_{exp,i}\right|}{x_{exp,i}}\right)$$

where *x_exp,i_* stands for the experimental values, and *x_pre,i_* denotes the predicted values obtained by calculating these measurements using the model. *N* and *n* are the number of observations and constants, respectively.

Moreover, the decimal reduction time (D time) was calculated for each combination of a tested value of heat or PAA. This value is defined as the contact time between bacterial cells and the killing agent required for decreasing bacterial concentrations at 1 log CFU. As a crucial parameter used to quantify the influence of the killing agent on the tested microorganism, it represents the time it takes to reduce the population by 90% (or one log cycle) at the tested killing agent value [47].

For the evaluation of the synergistic effect of heat and PAA concentrations, several mathematical steps were involved. All experiments were part of the previously described Box–Benken experimental design using the assumed minimal, optimal, and maximal values of heat and PAA concentrations. Firstly, the obtained results of this experimental design are presented using the response surface method (Minitab^®^ 21 Statistical Software). The axes of these graphs show PAA concentrations and temperature values, while the third variable (contact time) was a constant in the central value (3 min). The relationships between independent factors and the system’s response (*Y*) are calculated using a second-order polynomial equation (*Y*), where *b*_0_ is the intercept; and *b*_i_, *b*_ii_, and *b*_ij_ are the linear, quadratic, and regression coefficients, respectively. *X*_i_ and *X*_j_ represent the varied independent factors of the system. The individual effects of variables and their interactions were evaluated based on their *p*-values (α < 0.05).

(6) $$Y_{k}=b_{0}+\sum_{i=1}^{3}b_{i}\cdot X_{i}+\sum_{i=1}^{3}b_{\mathrm{ii}}\cdot X_{i}^{2}+\sum_{i=1,j=i+1}^{3}b_{\mathrm{ij}}\cdot X_{i}\cdot X_{j}$$

As the target output, the bacterial concentration was taken into account. However, in order to define the percentage of damaged cells among the surviving cells, an indirect determination of the number was performed on both selective and non-selective media (XLD or TSA agar). In this way, the surviving cell concentration and percentage of damaged cells were calculated (Figure 2), and both were involved in the calculations. In brief, with respect to terminology, the viable cell possesses the capability to indefinitely propagate under suitable conditions. The viability of a cell is demonstrated by its ability to thrive, whether on a solid surface or in a liquid medium [48]. The main goal of food preservation methods is to modify bacterial cells in such a way that they are inactivated, i.e., inhibited by some injury. The described cells are called damaged (injured) cells, which do not have the ability to reproduce during the application of antimicrobial treatment but also do not die. This modification of cells during antimicrobial treatment consists of changing one or more cellular structures or functions [49].

## 3. Results

### 3.1. Effect of Heat on the Inactivation of Salmonella Cells

**Figure 3:**
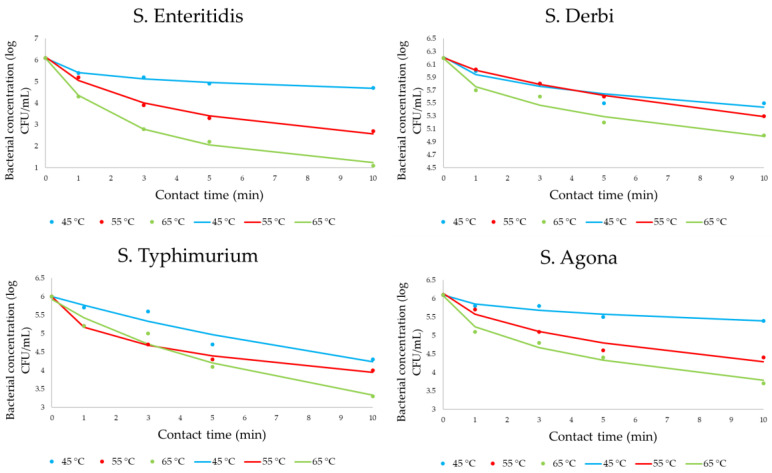
The four-parameter sigmoidal mathematical model for heat influence on *Salmonella* concentrations (dots are correlated with experimentally obtained results, while lines define the predictive response of kinetic models).

Figure 3 presents the obtained data for monitoring bacterial concentrations. Bacterial concentrations at zero minute presented initial concentration values without heat influence. All results are presented as experimentally obtained results (dots on graphs) and mathematically calculated results (lines on graphs).

The initial concentration for all serotypes was between 6 and 6.2 log CFU/mL. It is noticeable that there is a decrease in the number of all investigated cases. However, the decreasing trends not only differed significantly at the level of applied temperatures but also differed at the level of serotypes. The most sensitive strain to the effect of heat is *S. Enteritidis*, while the most resistant is *S. Derby*.

The regression coefficients and the standard errors of the obtained kinetic models are given in Table 3. All regression coefficients presented in the mentioned table are statistically significant at *p* < 0.05.

Table 4 illustrates the degree of agreement between experimental measurements and the model-calculated results. 

Using the obtained graphical reproduction of results of the individual effect of heat on bacterial concentration, decimal reduction times (D values) were calculated (Table 5). The minimum D values are 2.4, 4.65, 1.95, and 2.35 min for *S. Enteritidis*, *S. Derby*, *S. Typhimurium*, and *S. Agona*, respectively, and all were obtained at 65 °C. In the case of a temperature effect of 65 °C, the time required for the reduction of one log_10_ unit is below 2 min, and this was only observed for *S. Typhimurium*. A time of 2.8 min is necessary for the inactivation of the same concentration of this serotype at temperatures of 45 and 55 °C. For all other tested serotypes, the time necessary to reduce the defined cell concentration is greater than 6 min.

### 3.2. Effect of Peroxyacetic Acid on the Inactivation of Salmonella Cells

**Figure 4:**
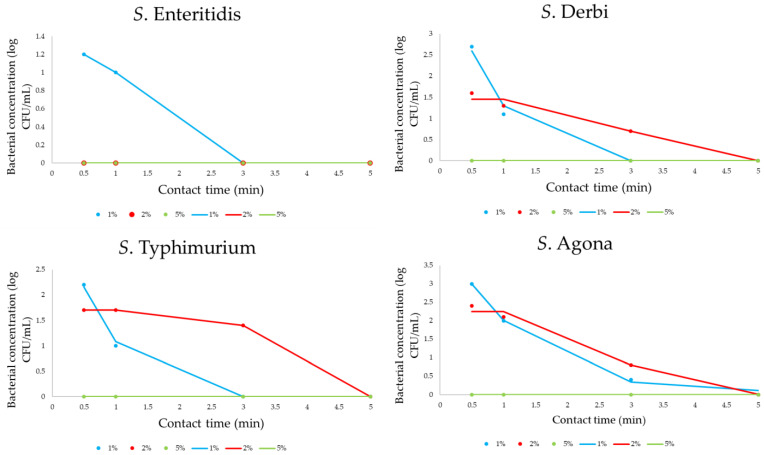
The four-parameter sigmoidal mathematical model for PAA influence on *Salmonella* concentration (dots are correlated with experimentally obtained results, while lines define the predictive response of kinetic models).

Figure 4 presents the obtained data for monitoring bacterial concentrations. Tested PPA solutions were prepared as 1, 2, and 5% solutions. In the conducted research study, the influence of different concentrations of peroxyacetic acid solution (1, 2, and 5%) on the inactivation of *Salmonella* serotype cells, for which their initial number was between 6 and 6.2 log CFU/mL, was examined. The contact time was also varied, and the short-term contact of cells with the disinfectant was examined for 30 s and 1, 2, 3, and 5 min. Figure 4 shows the results of the effect of the PAA solution during the selected contact time for the tested isolates. Based on these results, kinetic modeling was performed. All results are presented as experimentally obtained results (dots on graphs) and mathematically calculated results (lines on graphs).

The regression coefficients and the standard errors of the obtained kinetic models are given in Table 6. All regression coefficients presented in the mentioned table are statistically significant at *p* < 0.05.

Table 7 not only demonstrates the goodness of fit but also assesses the model’s quality via residual analyses. Crucially, the lack of significant lack-of-fit tests indicates that the models effectively represent the data. With the obtained parameters, the model comprehensively captures variations, showcasing a robust fit with the data.

As previously explained, decimal reduction times were also calculated for PAA’s influence on bacterial concentrations (Table 8). As observed for PAA influences, minimal D values are 0.1 min for all four *Salmonella* serotypes, indicating a strong influence of the 5% PAA solution. However, the D values for 1 and 2% PAA solution did not exceed 1.5 and 1.15 min, respectively.

### 3.3. Synergistic Effect of Heat and Peroxyacetic Acid on the Inactivation of Salmonella Cells in Clean Conditions

Table 9 summarizes the data of the Box–Benken design in clean conditions. The tested outputs were surviving cell concentration and the percentage of damaged cells obtained via incubation on non-selective and selective nutrient media, respectively. The effectiveness of disinfection treatment combining PAA with concentration, heat, and contact time variations indicated that the use of PAA, even at low concentrations, showed notable effectiveness in reducing *Salmonella* regardless of the serotype. However, the impact of heat on *Salmonella* inactivation was also evaluated, revealing its dependence on specific culture conditions. Generally, mild temperatures were associated with extended times required for significant inactivation, making them less feasible from an industrial perspective (Table 9).

**Figure 5:**
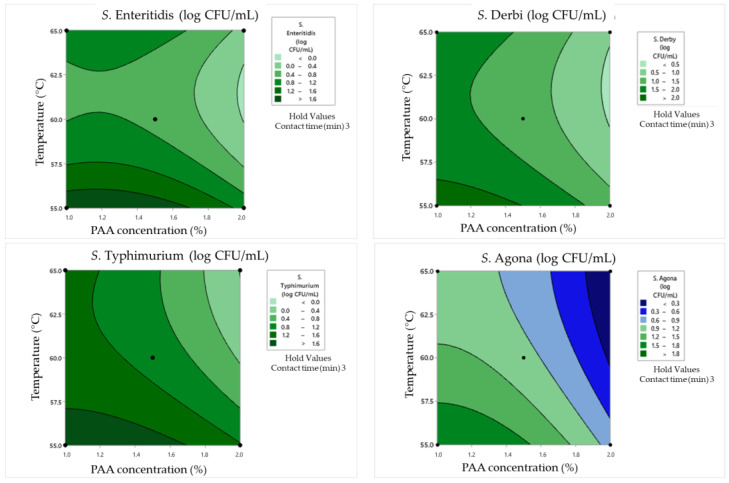
Response surface plots for the Box–Behnken experimental design in clean conditions for the concentration of surviving cells.

**Figure 6:**
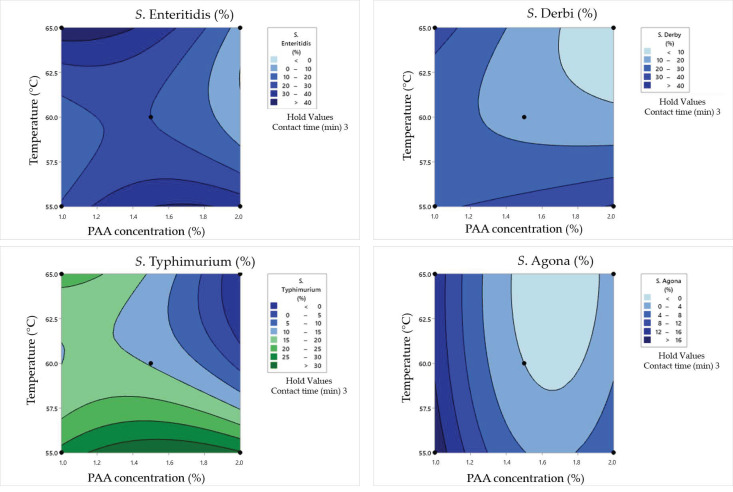
Response surface plots for the Box–Behnken experimental design in clean conditions for the cell damage rate.

The obtained data for the surviving *Salmonella* cell concentration in clean conditions was analyzed via response surface plots (Figure 5), which are consequently used for graphical optimization and the determination of the optimal values of examined parameters. The same evaluation protocol was repeated for damaged *Salmonella* cells in clean conditions (Figure 6).

In the case of the minimum tested contact time of 1 min, the obtained results underscored the substantial impact of PAA concentrations on *Salmonella* survival and damage rates. Namely, higher PAA concentrations, such as 2%, lead to more effective bacterial inactivation, resulting in lower surviving cell concentrations and reduced damage rates. On the other hand, the influence of temperature is not negligible. The highest tested temperatures tend to enhance the inactivation effect for all serotypes. For example, at 60 °C as the middle value, *S. Enteritidis* exhibited a surviving cell concentration of 1.56 log CFU/mL with a cell damage rate of 19%. When the temperature was increased to 65 °C, the same concentration of *S. Enteritidis* (2.01 log CFU/mL) was observed but with a lower damage rate (15%). The obtained graphical optimization also indicates that different *Salmonella* serotypes exhibit varying sensitivities to PAA and temperatures. *S. Typhimurium*, for instance, displayed a higher damage rate at 60 °C (30.5%) compared to other serotypes under the same conditions.

Longer contact times generally lead to lower surviving cell concentrations and higher damage rates. Using a contact time of 3 min, higher PAA concentrations also lead to more effective bacterial inactivation and reduced surviving cell concentrations as in the previously explained combinations. For example, when PAA concentrations increased from 1% to 2%, *S. Enteritidis* exhibited a significant reduction in surviving cell concentrations from 2.2 to 0.9 log CFU/mL, and cell damage rates varied from 27% to 25% after a 3 min contact time at 55 °C. Additionally, at higher temperatures (65 °C), bacterial inactivation was more pronounced. For instance, at 65 °C with 1% PAA, *S. Enteritidis* showed a surviving cell concentration of 1.23 log CFU/mL, whereas it was 2.2 log CFU/mL at 55 °C. The cell damage rate also increased from 27% to 59%. Furthermore, *S. Derby* showed higher damage rates at 55 °C compared to other serotypes under the same conditions.

In the case of a contact time of 5 min between 1% PAA and a temperature of 60 °C, *S. Enteritidis* exhibited complete inactivation (no surviving cells) with a cell damage rate of 0%. However, other serotypes, like *S. Typhimurium*, *S. Derby*, and *S. Agona*, exhibited varying levels of survival and damage after 5 min of the synergistic effect of tested influences. Doubling the PAA concentration to 2% at 60 °C led to a significantly reduced survival of *Salmonella*. Only *S. Derby* survived, albeit in a minimal concentration (0.3 log CFU/mL) and without any damaged cells. Temperature variations had different effects on different serotypes. For instance, when the temperature was lowered by 5 °C (55 °C instead of 60 °C) with 0.5% PAA, *S. Enteritidis* and *S. Typhimurium* showed increased survival compared to the 60 °C condition. This demonstrates that lower temperatures can reduce the effectiveness of PAA in certain cases. Even when some cells survive, the damage rates can differ significantly. For example, at 55 °C with 0.5% PAA, *S. Enteritidis* had a higher cell damage rate (54%) compared to *S. Typhimurium* (51%), indicating varying degrees of sensitivity relative to the disinfection process.

ANOVA analysis was conducted to assess the synergistic impact of temperature, time, and PAA concentrations using second-order polynomial models (SOPs) in clean conditions. The influence of these variable factors on response variables (SSE, SST, SSD, and SSA) was examined (refer to Table 10). The results indicated that the synergistic effect of temperature, time, and PAA concentrations primarily stemmed from the linear terms of SSE, SST, SSD, and SSA SOP models (refer to Table 10). Also, the quadratic temperature term significantly affected SOP calculations for SSE (*p* < 0.01) and SSD (*p* < 0.05). Additionally, the non-linear interaction term between PAA and time had a significant impact on SSD (*p* < 0.05), while the interaction between temperature and time significantly influenced SST (*p* < 0.05). The coefficients of determination (*r*^2^) for SOP models were notably high, ranging from 0.628 to 0.965 (refer to Table 10). Higher *r*^2^ values were associated with SOP models where non-linear terms exerted a stronger and more distinct influence. Despite this, the relatively imprecise results obtained from SOP models suggest the possibility of exploring alternative nonlinear models to enhance the accuracy of predictions.

### 3.4. Synergistic Effect of Heat and Peroxyacetic Acid on the Inactivation of Salmonella Cells in Dirty Conditions

**Figure 7:**
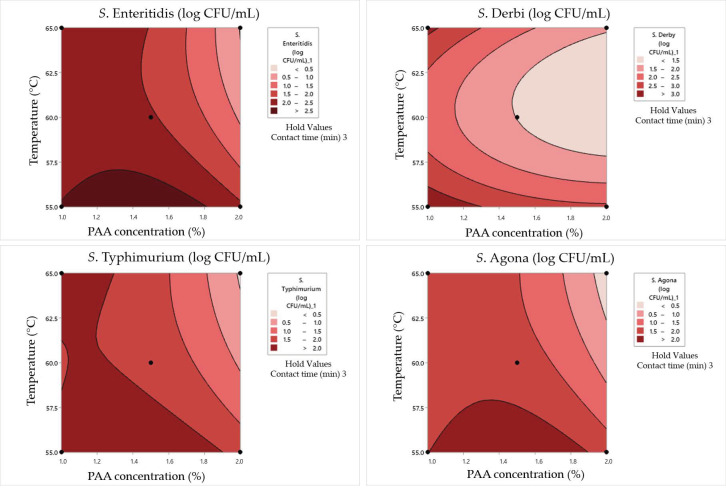
Response surface plots for the Box–Behnken experimental design in dirty conditions for the concentration of surviving cells.

**Figure 8:**
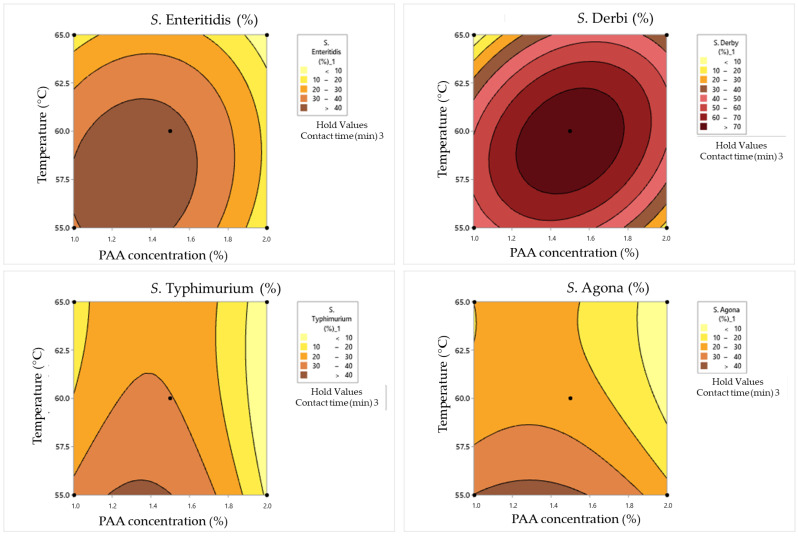
Response surface plots for Box–Behnken experimental design in dirty conditions for the cell damage rate.

Table 11 summarizes data on the Box–Benken experimental design in dirty conditions. Tested outputs were surviving cell concentrations and the percentage of damaged cells obtained via incubation on non-selective and selective nutrient media, respectively. The results revealed that under certain conditions, particularly at higher PAA concentrations and longer contact times, significant reductions in *Salmonella* survival were achieved. However, these reductions were accompanied by increased damage rates, indicating a trade-off between the effectiveness of inactivation and potential cellular damage. Therefore, the obtained data for surviving *Salmonella* cell concentrations in dirty conditions were analyzed via response surface plots (Figure 7), which were consequently used for graphical optimization and the determination of optimal values of examined parameters. The same evaluation protocol was carried out for cell damage rates in dirty conditions (Figure 8).

The combination of 1% PAA and a temperature of 60 °C for 1 min resulted in various survival and damage rates for different *Salmonella* serotypes. *S. Enteritidis* exhibited a moderate surviving cell concentration (1.88 log CFU/mL) with a damage rate of 16%, while *S. Typhimurium* showed a higher concentration (2 log CFU/mL) with a lower damage rate (7%). *S. Derby* and *S. Agona* had relatively high survival concentrations (2.65 and 1.45 log CFU/mL) but with varying damage rates (5% and 11%, respectively). Doubling the PAA concentration to 2% at the same temperature and contact time led to a significant reduction in survival, with only *S. Derby* surviving (0.48 log CFU/mL) but with a higher damage rate (33%). Increasing the temperature to 65 °C had a pronounced effect on survival and damage rates. For *S. Enteritidis*, the survival concentration decreased to 0.84 log CFU/mL, with no damaged cells. *S. Typhimurium* showed a slight decrease in survival (1.30 log CFU/mL) with a damage rate of 15%. *S. Derby* and *S. Agona* displayed substantial reductions in survival and varying damage rates under these conditions.

In the case of a contact time of 3 min, some pathways with respect to bacterial behavior can be summarized. In combination with 1% PAA at a temperature of 55 °C, *S. Enteritidis* demonstrated resilience, maintaining a relatively high survival concentration of 2.43 log CFU/mL. This can suggest that this serotype exhibits a certain degree of resistance to PAA at this concentration and temperature. *S. Typhimurium* displayed a similar pattern, with a survival concentration of 2.31 log CFU/mL. *S. Derby* exhibited remarkable robustness, surviving with a high concentration of 3.42 log CFU/mL, although significant cell damage was observed (61%). This highlights its exceptional resilience in the presence of low concentrations of PAA and elevated temperatures. *S. Agona* demonstrated moderate resistance, with a survival concentration of 2.24 log CFU/mL and 43% cell damage. It is more resistant than *S. Typhimurium* but less so than *S. Derby*. When the PAA concentration was increased to 2%, *S. Enteritidis* still showed the same level of survival, with a concentration of 2.21 log CFU/mL and a reduced cell damage rate of 17%. *S. Typhimurium*, *S. Derby*, and *S. Agona* exhibited reduced survival concentrations (1.91, 2.95, and 1.91 log CFU/mL, respectively) with lower cell damage rates (15%, 12%, and 21%, respectively) compared to the 1% PAA concentration. At a contact time of 3 min at 65 °C, *S. Enteritidis* displayed a survival concentration of 2.32 log CFU/mL with a 9% cell damage rate when subjected to 1% PAA. This suggests that elevated temperature enhances PAA’s effectiveness in reducing survival with low cell damage. Moreover, *S. Typhimurium* showed a similar trend at 65 °C, with reduced survival (2.12 log CFU/mL) and minimal cell damage (9%). *S. Derby* exhibited resistance, maintaining a survival concentration of 2.80 log CFU/mL with low cell damage (6%), while *S. Agona* displayed a moderate level of resistance, surviving at 1.68 log CFU/mL with a 23% cell damage rate. With a double PAA concentration (2%) and exposure to 65 °C, *S. Agona* showed no surviving cells, indicating high susceptibility to this combination. *S. Enteritidis* and *S. Typhimurium* exhibited minimal survival (0.6 and 0.3 log CFU/mL, respectively) with no cell damage. This suggests that high PAA concentrations combined with elevated temperatures can effectively eliminate these serotypes. Remarkably, *S. Derby* stood out as an exceptionally resistant serotype, maintaining a survival concentration of 2.02 log CFU/mL with a 12% cell damage rate.

When the maximum tested contact time of 5 min was combined with a 1% PAA concentration and a temperature of 60 °C, *S. Enteritidis* and *S. Typhimurium* displayed modest survival concentrations at approximately 1 log CFU/mL. However, their cell damage rates varied significantly, with *S. Enteritidis* experiencing a 30% damage rate and *S. Typhimurium* having a higher 40% damage rate. *S. Derby* and *S. Agona* maintained higher survival concentrations of 2.23 and 0.9 log CFU/mL, respectively, with cell damage rates of 12% and 13%. This suggests differential susceptibility to PAA treatment among *Salmonella* serotypes. When the PAA concentration was increased to 2% and the other two parameters were simultaneously maintained at the same values, *S. Enteritidis* was completely inactivated, while *S. Typhimurium*, *S. Derby*, and *S. Agona* exhibited varying degrees of survival ranging from 0.4 to 1 log CFU/mL. Notably, *S. Derby* displayed a substantial 40% cell damage rate. When the temperature was reduced by 5 °C, along with a PAA concentration (1.5%), a different outcome was observed. *S. Enteritidis* showed a higher survival concentration of 2.62 log CFU/mL with a 28% cell damage rate, while *S. Typhimurium* exhibited a survival concentration of 2.24 log CFU/mL with a 47% cell damage rate. *S. Derby* exhibited a higher survival concentration of 2.98 log CFU/mL with a 29% cell damage rate, and *S. Agona* displayed a concentration of 2.21 log CFU/mL with a substantial 51% cell damage rate. This suggests that lower temperatures and lower PAA concentrations can lead to higher survival concentrations and damage rates. In contrast, when a combination of 1.5% PAA and a temperature of 65 °C was applied for 5 min, all four strains—*S. Enteritidis*, *S. Typhimurium*, *S. Derby*, and *S. Agona*—exhibited consistent survival concentrations of approximately 2.62, 2.24, 2.98, and 2.21 log CFU/mL, respectively. Their cell damage rates were approximately 28%, 47%, 29%, and 51%, respectively.

An ANOVA analysis was conducted to evaluate the combined effects of temperature, time, and PAA concentrations using second-order polynomial models (SOPs). The impact of these factors on response variables (SSE, SST, SSD, and SSA) was examined, as shown in Table 12. The results revealed that the synergistic influence of temperature, time, and PAA concentrations primarily originated from their linear and quadratic terms (refer to Table 12). The coefficients of determination (*r*^2^) for SOP models were remarkably high, ranging from 0.833 to 0.968 (refer to Table 12). 

## 4. Discussion

### 4.1. Effect of Heat on the Inactivation of Salmonella Cells

The cell inactivation of four selected *Salmonella* serotypes via heat was included in the first step of this research study. A temperature of 45 °C could be defined as insufficiently effective for all tested bacteria, considering that the decreasing trend of the number of viable cells is minor. If the ability to maintain a certain temperature is minimal when in contact with a cold work surface, the previous observation is additionally supported. Heat treatment at a temperature of 55 °C caused a linear decrease in the total population of all tested isolates, but the overall treatment reduced the population number far less compared to the treatment at a temperature of 65 °C. The advantages of applying the highest tested temperature are also easily visible in the presented graphs, especially in the case of *S. Enteritidis* and *S. Typhimurium* where the abundance of the tested serotypes decreases sharply. Also, it is noticeable that *S. Derby* is the most resistant strain tested at a temperature of 65 °C (Figure 3). In the research conducted by Hassan et al. [50], a similar behavior was obtained in the case of *Salmonella* S1, S4, and S7 isolated from food samples. The mentioned isolates were almost completely resistant to the influence of a temperature of 55 °C, while a temperature of 65 °C was determined as efficient and effective for cell inactivation. Additionally, D_65 °C_ values were calculated, and the obtained values were 0.65, 1.02, and 0.49. This parameter enables the comparison of the relative heat resistance of different microorganisms. In the recent literature, this value for *S. Enteritidis* is between 1.3 and 2.7 min [51] at 55 °C. Low D values of 0.22 and 0.66 min at 65 °C were defined for *S. Typhimurium*, which is associated with dependence on the incubation temperature of the primary bacterial culture [52]. In comparison with the obtained results in Table 5, these results certainly indicate lower values. Conversely, for the thermal inactivation of *S. Agona* cells, a D parameter of 5.2 min at 65 °C is defined [53], which is significantly higher than the obtained values in this study. Rajkowska et al. [54] reported a significant difference in the D values obtained for heat treatments at 55 and 65 °C for *Salmonella* isolated from meat and seafood. Namely, D values for 55 °C were within the range of 7.08 and 7.5 min, while the same effect on cell inactivation at 65 °C was obtained for the range of 0.45–0.86 min. Summarizing the presented data, it can be concluded that the comparison of D values in research is extremely difficult due to the significant influence of various factors on thermal inactivation, such as the selected liquid diluent, temperature, bacterial strain, physiological state of microbial cells, heat exposure conditions, the possibility of cell recovery from sublethal injuries, etc. However, it has long been assumed that the relationship between the number of surviving cells and the time of exposure to constant temperature is exponential [38]. While deviations from traditional exponential inactivation kinetics and numerous exceptions exist, D values continue to serve as a valuable indicator for assessing heat resistance levels and facilitating comparisons among various microorganisms and experimental scenarios [48].

Other observations of the obtained results can be mentioned. It is often in the kinetic studies of heat-influenced cell inactivation that a so-called “shoulder” of the curve during the initial warm-up time is observed. This phenomenon indicates that cells are inactivated at a slower rate [55]. However, in this research study, the described occurrence was not observed, which potentially indicates the fast, uniform, and correct manipulation of the selected system for thermal inactivation and temperature constancy during the experiment. The obtained kinetic pathways are strong proof of this state, while additional calculations numerically confirm the quality of the presented mathematical modeling. The selected numerical verification parameters specify the simplicity, robustness, and accuracy of the presented four-parameter sigmoidal mathematical models. Importantly, the lack of fit tests for mathematical models proved to be insignificant, signifying their satisfactory representation of the data. It can be concluded that mathematically formed predictive models for all four *Salmonella* serotypes can be tools for the prediction of bacterial behavior during heat influence in tested conditions. Also, in some thermal inactivation tests, the formation of cell flocculates was visually observed, which was previously described in the scientific literature to be related to *S. Enteritidis* [56]. This clustering of cells is thought to have a protective effect on *Salmonella* cells during antimicrobial treatment. In the case of grouping, the cells in the water environment behave the same as when they are in the biofilm, showing greater resistance to the applied antimicrobial treatment. 

In summary, heat treatment using temperatures between 45 and 65 °C cannot be considered effective enough to achieve a targeted biocidal effect, considering that the contact time necessary for the reduction in a certain number of cells is extremely high. Therefore, it is necessary to examine other or additional influences of factors that would enable a greater reduction in cell numbers in the shortest possible period. Also, it should be emphasized that for almost an entire century, the food industry assumed that during the assessment of treatment outcomes, thermal inactivation always follows first-order kinetics. However, there is increasing evidence that supports the possibility that the inactivation of microbial cells does not follow this kinetic model, especially during mild heat treatment. In the case where the obtained curves are not log-linear, Cebrián et al. [48] emphasized that the D value as an efficiency criterion should be taken with special caution. Therefore, an alternative concept for thermal microbial inactivation is recommended and denoted as tkD, which was developed to describe the resistance of microorganisms to heat. This criterion describes the time (t) required for a reduction in k log units in the microbial population. In this concept, the deviation from first-order kinetics is taken into account when evaluating the efficiency of the thermal treatment. In the end, it should be emphasized that the size of the inoculum, i.e., the initial concentration of cells, was not considered in the presented research study because the most undesirable situation was taken into account—a large number of initially present cells. Further research would answer the hypothesis of whether the difference in the effect of heat can be easily observed with a lower-concentration inoculum of *Salmonella* species, i.e., faster inactivation of a log unit of cells compared to high-concentration inoculum experiments. 

### 4.2. Effects of Peroxyacetic Acid on the Inactivation of Salmonella Cells

Based on the linear dependence on the obtained models, it is noticeable that there is a decrease in the number in all examined cases. Also, higher sensitivities can be defined for *S. Enteritidis* and *S. Agona* for all tested concentrations compared to *S. Derby* and *S. Typhimurium*. A concentration of 5% could be defined as fully effective given that the reduction in the number of viable cells is almost instantaneous. However, the 2% solution proved to be very effective against *S. Enteritidis*, for which its effect was comparable to the highest concentration tested. Although decreasing trends are also observed for 1% PAA treatment for all bacteria, different kinetic pathways are obtained compared with the effect of the previously mentioned PAA concentration. For all tested isolates, during contact with the 5% PAA solution, less than 0.1 min is necessary for a reduction of one log10 unit. The same D value was obtained for *S. Enteritidis* upon contact with a 2% solution, while the D values were between 0.8 and 1.15 min for other tested serotypes. The highest D values were observed when applying a 1% PAA solution; thus, for the reduction of one log10 unit, a minimum of 0.7 min was necessary for *S. Enteritidis*, and a maximum of 1.5 min was necessary for *S. Derby*. As the current use of PAA as a disinfectant solution is still limited, far fewer studies have been conducted compared to thermal inactivation studies. Aryal and Muriana [57] defined a D value of 1.67 min as sufficiently effective for the logarithmic reduction in *S. Montevideo* with a 1% PAA solution. As part of the research by Kumar et al. [58], the D value for S. *Typhimurium* is 1.76 min when applying a 0.05% solution. For the same serotype, a D value of 2.02 min was also demonstrated when cells were contacted with a 0.04% solution [59]. It is noticeable that lower concentrations of the tested solution were used in the mentioned research; however, the chosen concentrations of the disinfectant in this research study are in accordance with the manufacturer’s recommendations. More specifically, PAA solutions in concentrations between 10% and 15% represent stable forms for industrial storage over a long period, and the long-term storage of solutions lower than 1% is not recommended [60]. The presented data not only demonstrate the goodness of fit of kinetic models but also assess the model’s quality via residual analyses. Crucially, the lack of significant lack-of-fit tests indicates that the models effectively represent the data. Numerical verification shows a robust fit with the data. It can be concluded that mathematically formed predictive models for all four *Salmonella* serotypes can be tools for the prediction of bacterial behavior during PAA influence in tested conditions.

In summary, antimicrobial treatment using PAA solution concentrations between 1 and 5% can be considered effective enough to achieve a biocidal effect given that the contact time necessary for the reduction of one log10 unit of cells is equal to or less than 1.5 min. In view of the ecological and economic criteria, saving chemical agents is necessary at the industrial level, and lower concentrations are most often applied. Therefore, PA concentrations of 1 and 2% can be singled out as effective for applications. In addition to this, the processes of sanitation and disinfection are often coupled, and it is necessary to examine the mutual influence of the treatment, which would potentially enable a reduction in economic parameters in the case of the possibility of using lower concentrations of disinfectants, lower temperatures, etc. An additional observation during the PAA cell inactivation treatment study is the fact that there was no formation of cell flocculates visible to the naked eye in contrast to the heat inactivation assay, which potentially indicates that the cells do not have enough time to activate any defense mechanism.

One more essential observation needs to be addressed. Bacterial growth temperatures are a critical factor that can significantly influence the resistance of *Salmonella* to stressors such as antimicrobial agents [58]. This study not only included *Salmonella* growth at 37 °C as the optimal growth temperature but also the average body temperature for many mammals. Consequently, the optimal growth conditions can lead to metabolic balances without a timely response to environmental stress. On the other hand, when *Salmonella* is cultured at a lower temperature, it might exhibit different responses to stress [58]. A parallel hypothesis can be posited for bacterial resistance in the context of PAA influences. The reasons for such responses in bacterial behavior can be explained at the metabolic level since stress response proteins during *Salmonella* grown at a temperature below 20 °C appear to be more selective in maintaining the proper folding of proteins and the protection of mRNA. Many proteins involved in amino acid metabolism, virulence proteins, and functional flagella formation can be affected during cold stress at low *Salmonella* growth temperatures [58]. An additional reason for further investigation in this direction is the used temperature range in the meat industry because many steps during meat processing involve refrigeration temperatures. The proposed direction is an investigation of *Salmonella* resistance against heat and PAA, which could be of principal importance, offering insights into the temperature-dependent characteristics of distinct serotypes. The anticipated outcomes may include the formulation of more stringent disinfection protocols and the enhancement of overall hygienic practices in food processing.

Investigating how growth temperatures affect *Salmonella* resistance to heat and PAA can be crucial and lead to an understanding of the growth temperature dependence of the used serotypes. As an outcome, strict temperature control and better hygienic practices can be expected. 

### 4.3. Synergistic Effects of Heat and Peroxyacetic Acid on the Inactivation of Salmonella Cells in Clean Conditions

Recognizing that a single-pronged reliance on heat or peroxyacetic acid for tested *Salmonella* inactivation might not be the most maintainable strategy, a comprehensive Box–Behnken evaluation of the synergistic effect of heat and peroxyacetic acid on four *Salmonella* serotypes was carried out for the first time. In clean conditions, the minimum number of surviving cells is achieved at temperatures between 60 and 62.5 °C, while the concentration of the PAA solution has the greatest influence at the highest tested value. In the case of *S. Typhimurium*, the minimum number of surviving cells was reached at temperatures from 60 to 65 °C when the concentration of the PAA solution was within the range of 1.5% to 2%. The obtained plots also show the prediction of the number of surviving cells after antimicrobial treatment for *S. Derby* and *S. Agona*. Based on the presented results, it was clearly observed that in the case of *S. Derby*, the number of surviving cells decreased directly with respect to an increase in PAA solution concentrations and temperatures. In contrast, the lowest number of *S. Agona* surviving cells was already observed at a temperature of 60 °C and a PAA solution concentration within the range of 1.5 to 2%. The percentage of damaged cells for *S. Enteritidis*, *S. Typhimurium*, *S. Derby*, and *S. Agona* is within the range of 0–59, 0–51, 0–50, and 0–30%, respectively, which indicates the importance of testing this parameter. Achieving a high percentage of damaged cells in the population of surviving *S. Enteritidis* cells requires the maximum investigated temperature value and the minimum PAA solution value. Also, the figure shows a minimum region for a high percentage of damaged cells at a temperature of 55 °C in the case of medium concentrations of the PAA solution. The research by Suo et al. [58] proved that when mild heat treatment (55 °C) was used, significant sublethal injuries occurred, the percentage of which decreased over time. On the other hand, in the case of *S. Typhimurium*, the maximum percentages of damaged cells were obtained in the case of the minimum temperature at almost the entire concentration range of the PAA solution. The highest number of damaged cells of *S. Derby* was observed at the minimum and maximum tested temperatures and almost in the entire range of tested PAA solution concentration values. On the other hand, the percentage of damaged *S. Agona* cells is the highest in the case of low-temperature values and low PAA solution concentrations, but the relative percentage of damaged cells is much lower compared to all other examined serotypes.

Based on the ANOVA calculations, it can be noted that all three tested variables have a significant impact on the survival of all tested serotypes (bold results for individual parameters). However, the effects of the interactions among the variables differ at the serotype level. For surviving *S. Enteritidis* cells (SSEs), the greatest influence was observed with respect to temperature, while the mutual influence of temperature and contact time was the most significant for surviving *S. Typhimurium* cells (SSTs). In the case of surviving *S. Derby* cells (SSDs), the interrelationships between temperature and contact times and the concentration of the PAA solution and contact times have a significant effect on the number of surviving cells. On the other hand, with surviving *S. Agona* cells (SSA), no significant dependence on the mutual influence of investigated variables was observed. Given the observed serotype, *S. Derby* showed the greatest resistance in the examination of the single-factor effects of heat, i.e., peroxyacetic acid; the obtained results exhibited significant dependence on all examined factors, and this was expected. Stronger antimicrobial treatment is necessary for this strain given that the obtained D parameters are high compared to other serotypes; thus, the examination of the synergistic effect is of particular importance for this serotype. In order to confirm the described results, the same calculations were carried out for damaged cells. The calculated statistical parameters indicated the significance of the study of individual and mutual influences of antimicrobial treatment parameters. Interestingly, there are no effects of the system that stand out for the percentage of damaged *S. Enteritidis*, *S. Typhimurium*, and *S. Agona* cells (DSE, DST, and DSA), while for damaged *S. Derby* cells (DSD), two effects of particular importance were observed. In particular, for *S. Derby*, the effects of temperature, as well as the mutual relationship between the concentration of the PAA solution and temperature, are distinguished.

In summary, the synergistic effect of the combination of temperature and PAA solution concentrations had different efficacies on surviving and damaged cell populations of *Salmonella* serotypes under clean conditions. It can be assumed that during mild thermal treatment, cells are damaged over time, but they are not dead. When bacteria are exposed to heat stress, heat shock proteins are rapidly synthesized, help cell survival, and create a protective oxidative stress effect [60]. However, with the application of the examined values of PAA solution concentrations, it is possible to additionally influence the more efficient response of the system, and the following antimicrobial treatment values in clean conditions are generally recommended: temperatures higher than 60 °C, concentrations of PAA solution from 1.5 to 2%, and contact time of 3 min or more.

### 4.4. Synergistic Effects of Heat and Peroxyacetic Acid on the Inactivation of Salmonella Cells in Dirty Conditions

By comparing the number of surviving cells in clean and dirty conditions, the maximum values of surviving cells were obtained and were 19, 17, 37, and 20% higher relative to *S. Enteritidis*, *S. Typhimurium*, *S. Derby*, and *S. Agona*, respectively. From these data alone, it can be assumed that in dirty conditions, more aggressive antimicrobial treatment is needed to achieve the disinfection effect. The lowest number of surviving *S. Enteritidis* and *S. Typhimurium* cells is at temperatures of 60–65 °C and PAA solution concentrations between 1.5 and 2%. Achieving the maximum effect with *S. Derby* is not possible within the limits shown in the figure. This result confirms previous results that show that *S. Derby* is the most resistant strain and the potential need for antimicrobial treatments to last longer if the temperature and concentration values of the PAA solution are kept within the tested ranges. This is also evidenced by the obtained minimum surviving cell values in the experiment in which a contact time of 5 min was applied. Temperatures that are close to the tested maximum are necessary for the minimum number of surviving *S. Agona* cells, which also applies to the PAA solution’s concentration. Although the tested strains belong to the same species, each exhibits different resistance phenomena to antimicrobial treatment; thus, treatment parameters must be optimized individually for each serotype regardless of their phylogenetic similarity. However, common ranges can be defined for experiments under clean conditions: temperatures within the range of 62.5 to 65 °C, PAA solution concentrations between 1.5 and 2%, and contact times higher than 3 min. 

As in the case of the number of surviving cells, it is also possible to observe different percentages of damaged cells in relation to clean conditions. A lower percentage of damaged cells was obtained for *S. Enteritidis*, and the same was obtained for *S. Typhimurium*; a higher percentage was obtained in the case of the two remaining serotypes: *S. Derby* and *S. Agona*. A higher percentage of damaged cells in dirty conditions could have been the starting hypothesis given that the resistance of *Salmonella* cells in the presence of organic loads is higher [41], making the antimicrobial treatment insufficiently effective in causing cell death. However, the obtained results indicate differences at the serotype level. The maximum number of damaged cells is obtained at relatively low tested temperatures and concentration values with respect to the PAA solution in the case of *S. Enteritidis*. On the other hand, a high percentage of damaged *S. Typhimurium* cells was observed at a temperature of 55 °C and medium concentrations of the PAA solution. The obtained results of the *S. Derby* test indicate that the highest percentage of damaged cells is obtained by applying the mean values of the tested parameters. This is visible on the graph with cyclical circles that indicate the highest values at the center of the graph. In contrast, with respect to *S. Agona*, it is clearly observed that the application of the minimum temperature and moderate concentration values of the PAA solution is crucial for obtaining a higher percentage of damaged cells. Based on the presented results, it can be concluded that the application of the highest PAA solution concentration and temperature values during a contact time of 3 min or more is necessary in order to justify the effectiveness of antimicrobial treatment for all serotypes. 

The additional consideration of the influence of tested parameters on the response of the system was carried out via the ANOVA statistical processing of the obtained values of surviving cells in dirty conditions. The concentration of the PAA solution had the greatest individual influence on all tested serotypes. In the case of *S. Enteritidis* (SSE), additional influences of temperature as an individual factor and contact time as a mutual influence were observed. Individual influences of temperature, the mutual influence of PAA solution concentrations, and contact time, as well as PAA concentration and contact times, were confirmed for *S. Typhimurium* (SST). Unlike *S. Derby* (SSD), for which its response is additionally only influenced by the mutual influence of temperature, with respect to *S. Agona* (SSA), a significant influence of almost all mutual interactions of antimicrobial treatment parameters was recognized. 

The same calculation was carried out for the damage cell rate, which indicates that there is no uniformity in terms of which factor is the most significant on the effectiveness of antimicrobial treatment. The damage rate in the case of *S. Enteritidis* (DSE) is mostly affected by the individual and mutual influences of the concentration of the PAA solution and temperature. Equally important is the mutual influence of the contact time on the mentioned bacteria. The individual contribution of PAA solution concentrations and the overall effect of contact time is noticeable for *S. Typhimurium* (DST). The damaged *S. Derby* (DSD) is affected by mutual relations between the same individual parameters, while the percentage of *S. Agona* cell damage (DSA) is most positively influenced by temperature. In some antimicrobial treatment tests carried out in dirty conditions, cell flocs were observed, in addition to testing the effect of the thermal inactivation of cells. There is a possibility that the present organic load limited the antimicrobial effect, preventing the full effect of the active component of the chemical agent. This may also have contributed to the difference in the percentage of damaged cells during the study. In the end, it remains an open question whether cells trapped on the surface of the system are crucial for predicting the effect of antimicrobial treatments, especially from the point of view of the application of mild heat treatment.

## 5. Conclusions

This study proposes a comprehensive research concept in establishing an effective combination of growth-suppressing agents for applications in real processing conditions in the food industry. Since differences between clean and dirty conditions were evaluated via the Box–Behnken experimental design, the obtained results are a good basis for defining the disinfection protocol of industrial surface treatments. As a summary response of the tested synergistic strategies, the following varying factor values are recommended: temperatures from 60 to 65 °C, 1.5% peroxyacetic acid concentration, and contact times greater than 3 min. The data illustrate a complex relationship between peroxyacetic acid concentrations, temperatures, contact times, and the effectiveness of *Salmonella* inactivation. Achieving a balance between reducing pathogen survival and minimizing cellular damage is essential. In view of implementation in the industry, a procedure for maintaining the hygiene of facilities, equipment, and workspace, as well as accompanying documentation, can be formed, which will contribute to the implementation of disinfection procedures in industrial conditions. Additionally, further research could explore variations in disinfection protocols tailored to specific food industry sectors, offering a more targeted approach to enhancing microbial safety. On the other hand, enhancing the scientific understanding of microbial physiological factors associated with heat and chemical agent influences is imperative in order to prevent resistance and conditions that promote cell survival and recovery. The exact mechanisms of this synergistic effect are still under investigation, but they likely involve a combination of peroxyacetic acid, which breaks down the protective barriers of the bacteria, and heat, accelerating these processes. Understanding the physiological and biochemical aspects of this synergy will be important for optimizing disinfection procedures. In future studies, understanding complex microbial responses to heat and peroxyacetic acid exposure is fundamental to optimizing synergistic strategies in disinfection procedures.

## Figures and Tables

**Table 1:** Bacterial strains in the study.

Code of the Strains	Serotype	Location of Isolation
A1	*Salmonella Enteritidis*	Livestock slaughter and cutting department (slaughterhouse sector A)
A6	*S. Derby*	Livestock slaughter and cutting department (slaughterhouse sector A)
B1	*S. Typhimurium*	Stomach and intestinal cleansing department (slaughterhouse sector B)
B4	*S. Agona*	Stomach and intestinal cleansing department (slaughterhouse sector B)

**Table 2:** Experimental design of the operational parameters of antimicrobial treatment.

Experiment No.	Coded Values *			Numeric Values		
	*X*_1_	*X*_2_	*X*_3_	PAA Concentration (%)	Temperature (°C)	Contact Time (min)
1	−1	−1	0	1	55	3
2	1	−1	0	5	55	3
3	−1	1	0	1	65	3
4	1	1	0	5	65	3
5	−1	0	−1	1	60	1
6	1	0	−1	5	60	1
7	−1	0	1	1	60	5
8	1	0	1	5	60	5
9	0	−1	−1	1.5	55	1
10	0	1	−1	1.5	65	1
11	0	−1	1	1.5	55	5
12	0	1	1	1.5	65	5
13	0	0	0	1.5	60	3
14	0	0	0	1.5	60	3
15	0	0	0	1.5	60	3

* Each variable is coded with −1, 0, and 1 corresponding to the lowest, middle, and highest levels of variation, respectively.

**Table 3:** Regression coefficients (d, a, c, and b) and the standard errors of the four-parameter sigmoidal mathematical model for heat influence on *Salmonella* concentrations.

Serotype	*S. Enteritidis*			*S. Derbi*			*S. Typhimurium*			*S. Agona*		
Temperature (°C)	45	55	65	45	55	65	45	55	65	45	55	65
d	0.001 ± 0.000	0.001 ± 0.000	0.001 ± 0.000	0.001 ± 0.000	0.001 ± 0.000	0.001 ± 0.000	0.001 ± 0.000	0.001 ± 0.000	0.001 ± 0.000	0.001 ± 0.000	0.001 ± 0.000	0.001 ± 0.000
a	6.099 ± 0.006	6.141 ± 0.004	6.085 ± 0.006	6.214 ± 0.006	6.204 ± 0.008	6.194 ± 0.003	6.005 ± 0.010	6.007 ± 0.006	5.929 ± 0.005	6.094 ± 0.009	6.135 ± 0.003	6.074 ± 0.008
c	232.710 ± 19.237	6.618 ± 0.016	2.536 ± 0.001	482.502 ± 51.734	110.423 ± 2.009	172.619 ± 2.464	23.934 ± 2.246	36.232 ± 1.549	13.058 ± 0.176	621.091 ± 7.072	37.867 ± 0.472	24.060 ± 0.264
b	0.383 ± 0.004	0.807 ± 0.010	0.990 ± 0.000	0.501 ± 0.003	0.732 ± 0.006	0.499 ± 0.011	0.998 ± 0.022	0.506 ± 0.001	0.926 ± 0.009	0.494 ± 0.003	0.631 ± 0.004	0.577 ± 0.009

**Table 4:** The “goodness of fit” of the obtained four-parameter sigmoidal mathematical model for the individual effect of heat on *Salmonella* concentrations.

Serotype	Temp. (°C)	*χ* ^2^	RMSE	MBE	MPE
*S. Enteritidis*	45	0.002	0.043	0.000	0.644
	55	0.018	0.120	0.003	3.063
	65	0.011	0.094	−0.006	4.293
*S. Derbi*	45	0.007	0.076	0.000	1.113
	55	0.000	0.012	0.000	0.175
	65	0.007	0.075	0.000	1.061
*S. Typhimurium*	45	0.037	0.173	0.000	2.623
	55	0.003	0.051	0.000	0.923
	65	0.036	0.171	−0.001	2.898
*S. Agona*	45	0.005	0.066	0.000	0.899
	55	0.017	0.118	0.000	1.962
	65	0.013	0.100	−0.001	1.991

*χ*^2^, reduced chi-square; RMSE, root mean square error; MBE, mean bias error; MPE, mean percentage error.

**Table 5:** Decimal reduction time for the effect of heat.

D Time (min)	Serotype			
	*S. Enteritidis*	*S. Derby*	*S. Typhimurium*	*S. Agona*
D_45 °C_	>6	7.4	2.8	>7.2
D_55 °C_	3.3	5.9	2.8	3.1
D_65 °C_	2.4	4.65	1.95	2.35

**Table 6:** Regression coefficients (d, a, c, and b) and the standard errors of the obtained models for PAA influence on *Salmonella* concentrations.

Serotype	*S. Enteritidis*			*S. Derbi*			*S. Typhimurium*			*S. Agona*		
PAA concentration (%)	1	2	5	1	2	5	1	2	5	1	2	5
d	0.000 ± 0.000	0.000 ± 0.000	0.000 ± 0.000	0.000 ± 0.000	0.000 ± 0.000	0.000 ± 0.000	0.000 ± 0.000	0.000 ± 0.000	0.000 ± 0.000	0.000 ± 0.000	0.000 ± 0.000	0.000 ± 0.000
a	1.203 ± 0.005	0.000 ± 0.000	0.000 ± 0.000	2.600 ± 0.004	1.450 ± 0.000	0.000 ± 0.000	2.160 ± 0.001	1.700 ± 0.002	0.000 ± 0.000	3.413 ± 0.005	2.250 ± 0.009	0.000 ± 0.000
c	1.249 ± 0.011	1.000 ± 0.003	1.000 ± 0.002	1.000 ± 0.006	2.988 ± 0.010	1.000 ± 0.019	1.000 ± 0.025	3.307 ± 0.042	1.000 ± 0.028	1.167 ± 0.009	2.842 ± 0.0210	1.000 ± 0.0290
b	7.158 ± 0.019	7.146 ± 0.007	7.146 ± 0.014	36.178 ± 0.048	17.393 ± 0.163	7.146 ± 0.082	24.153 ± 0.083	15.800 ± 0.048	7.146 ± 0.071	2.33 ± 0.010	10.966 ± 0.042	7.146 ± 0.017

**Table 7:** The “goodness of fit” of the four-parameter sigmoidal mathematical model for PAA concentrations with respect to *Salmonella* concentrations.

Serotype	PAA (%)	χ^2^	RMSE	MBE	MPE
*S. Enteritidis*	1	0.000	0.001	−0.001	0.030
	2	0.000	0.000	0.000	0.000
	5	0.000	0.000	0.000	0.000
*S. Derbi*	1	0.017	0.112	−0.025	5.471
	2	0.015	0.106	0.000	5.232
	5	0.000	0.000	0.000	0.000
*S. Typhimurium*	1	0.003	0.045	−0.010	2.455
	2	0.060	0.212	0.000	9.120
	5	0.000	0.000	0.000	0.000
*S. Agona*	1	0.005	0.063	−0.014	3.888
	2	0.015	0.106	−0.002	3.386
	5	0.000	0.000	0.000	0.000

*χ*^2^, reduced chi-square; RMSE, root mean square error; MBE, mean bias error; MPE, mean percentage error.

**Table 8:** Decimal reduction time for the effect of the PAA solution.

D Time (min)	Serotype			
	*S. Enteritidis*	*S. Derby*	*S. Typhimurium*	*S. Agona*
D_1%_	0.7	1.5	1.45	0.95
D_2%_	0.1	0.95	1.15	0.8
D_5%_	0.1	0.1	0.1	0.1

**Table 9:** The Box–Behnken experimental data in clean conditions.

Box–Behnken Design			Surviving Cell Concentration(log CFU/mL)				Cell Damage Rate(%)			
PAA Concentration (%)	Temperature (°C)	Contact Time (min)	*S. Enteritidis*	*S. Typhimurium*	*S. Derby*	*S. Agona*	*S. Enteritidis*	*S. Typhimurium*	*S. Derby*	*S. Agona*
1	55	3	2.2	1.87	2.48	1.84	27	22	30	21
2	55	3	0.9	1.4	1.26	0.7	25	20	50	0
1	65	3	1.23	1.175	1.84	1.12	59	33	26	23
2	65	3	0	0	0.4	0	0	0	0	0
1	60	1	1.56	1.57	2.21	1.21	19	30.5	28	19
2	60	1	0.85	0.91	1.84	0.78	28.5	25	19	30
1	60	5	0	1.71	1.91	1.3	0	6	29	16
2	60	5	0	0	0.3	0	0	0	0	0
1.5	55	1	2.05	1.98	2.5	1.86	37	45	41	8
1.5	65	1	2.01	1.96	2.43	1.63	22	26	15.5	5
1.5	55	5	1.93	1.77	2.33	1.4	54	51	17	20
1.5	65	5	0.47	0.3	1.68	0	50	0	29	0
1.5	60	3	0.7	1.08	1.26	1.01	20	17	17	0
1.5	60	3	0.7	1.0	1.26	1.0	20	10	17	0
1.5	60	3	0.7	1.08	1.26	1.0	20	17	17	0

**Table 10:** ANOVA calculation of the synergistic effect of heat and PAA concentrations in clean conditions (sum of squares is shown).

Factor	df	SSE	SST	SSD	SSA	DSE	DST	DSD	DSA
PAA concentration	1	1.31 *	2.02 **	2.69 **	1.99 **	331.53	270.28	242.00	300.13
Temperature	1	1.42 *	1.61 **	0.62 *	1.16 **	18.00	780.13	569.53 *	55.13
Time	1	2.07 **	0.87 **	0.95 **	0.97 **	0.78	603.78	101.53	84.50
PAA concentration × PAA concentration	1	0.37	0.15	0.17	0.22	411.94	112.54	7.63	333.23
Temperature × Temperature	1	1.80 **	0.24	0.76 *	0.09	1238.21	340.58	240.01	8.31
Time × Time	1	0.17	0.14	1.01 **	0.01	21.94	143.27	1.17	168.23
PAA concentration × Temperature	1	0.00	0.12	0.01	0.00	812.25	240.25	529.00 *	1.00
PAA concentration × Time	1	0.13	0.28	0.38 *	0.19	22.56	0.06	100.00	182.25
Temperature × Time	1	0.50	0.53 *	0.08	0.34	30.25	256.00	351.56	72.25
Error	5	0.50	0.27	0.24	0.29	1775.31	675.60	340.06	468.25
*r* ^2^		0.940	0.957	0.965	0.946	0.628	0.804	0.863	0.713

Surviving cells concentrations for the following: SSE—*S. Enteritidis*; SST—*S. Typhimurium*; SSD—*S. Derby*; SSA—*S. Agona*. Cell damage rate for the following: DSE—*S. Enteritidis*; DST—*S. Typhimurium*; DSD—*S. Derby*; DSA—*S. Agona*. ** Statistically significant at *p* ≤ 0.01 level; * statistically significant at *p* ≤ 0.05 level.

**Table 11:** Box–Behnken experimental data in dirty conditions.

Box–Behnken Design			Surviving Cells Concentration(log CFU/mL)				Cell Damage Rate(%)			
PAA Concentration (%)	Temperature (°C)	Contact Time (min)	*S. Enteritidis*	*S. Typhimurium*	*S. Derby*	*S. Agona*	*S. Enteritidis*	*S. Typhimurium*	*S. Derby*	*S. Agona*
1	55	3	2.43	2.31	3.42	2.24	41	35	61	43
2	55	3	2.21	1.91	2.95	1.91	17	15	12	21
1	65	3	2.32	2.12	2.80	1.68	9	9	6	23
2	65	3	0.60	0.30	2.02	0.00	0	0	12	0
1	60	1	1.88	2.00	2.65	1.45	16	7	5	11
2	60	1	0.00	0.00	0.48	0.00	0	0	33	0
1	60	5	1.00	1.00	2.23	0.90	30	40	12	13
2	60	5	0.00	1.00	0.40	0.90	0	0	40	0
1.5	55	1	1.48	1.41	2.21	1.34	13	27	9	14
1.5	65	1	0.84	1.30	1.51	1.21	0	15	34	0
1.5	55	5	2.62	2.24	2.98	2.21	28	47	29	51
1.5	65	5	1.48	1.41	2.21	1.34	23	51	13	23
1.5	60	3	2.05	1.84	1.49	1.74	43	30	78	25
1.5	60	3	2.05	1.84	1.49	1.74	43	30	78	24
1.5	60	3	2.05	1.84	1.49	1.74	43	30	78	25

**Table 12:** ANOVA calculation for the synergistic effect of heat and PAA concentrations in dirty conditions (sum of squares is shown).

Factor	df	SSE	SST	SSD	SSA	DSE	DST	DSD	DSA
PAA concentration	1	2.90 *	2.23 **	3.45 *	1.50 **	793.33 **	722.58 *	17.82	575.20
Temperature	1	1.01	0.55 *	0.25	0.92 *	874.80 **	1396.76 **	2681.51 *	346.14
Time	1	1.54 *	0.94 **	1.14	1.50 **	555.68 *	297.12	267.94	858.44 *
PAA concentration × PAA concentration	1	0.48	0.16	4.04 *	0.17	418.02 *	51.50	2929.56 *	152.80
Temperature × Temperature	1	0.10	0.11	0.12	0.23	341.56 *	966.27 **	21.85	487.74
Time × Time	1	2.41 *	0.77 **	0.35	0.68 *	937.32 **	2.64	2940.71 *	325.61
PAA concentration × Temperature	1	0.56	0.51 *	0.02	0.46	59.99	30.20	753.87	0.43
PAA concentration × Time	1	0.19	1.00 **	0.03	0.53 *	50.27	272.25	0.24	0.99
Temperature × Time	1	0.06	0.13	0.00	0.13	18.90	63.05	409.94	57.62
Error	5	1.00	0.21	1.43	0.36	206.38	230.70	1779.53	487.68
*r* ^2^		0.903	0.968	0.869	0.944	0.948	0.944	0.833	0.853

Surviving cells concentration for the following: SSE—*S. Enteritidis*; SST—*S. Typhimurium*; SSD—*S. Derby*; SSA—*S. Agona*. Cell damage rates for the following: DSE—*S. Enteritidis*; DST—*S. Typhimurium*; DSD—*S. Derby*; DSA—*S. Agona*. ** Statistically significant at *p* ≤ 0.01 level; * statistically significant at *p* ≤ 0.05 level.

## Data Availability

Data are contained within the article. The data used to support the findings of this study can be made available by the corresponding author upon request.

## References

[1] Kumar D., Kalita P. (2017). Reducing Postharvest Losses during Storage of Grain Crops to Strengthen Food Security in Developing Countries. Foods.

[2] Newell D.G., Koopmans M., Verhoef L., Duizer E., Aidara-Kane A., Sprong H., Opsteegh M., Langelaar M., Threfall J., Scheutz F. (2010). Food-borne diseases—The challenges of 20 years ago still persist while new ones continue to emerge. Int. J. Food Microbiol..

[3] Bintsis T. (2017). Foodborne pathogens. AIMS Microbiol..

[4] Alegbeleye O.O., Singleton I., Sant’Ana A.S. (2018). Sources and contamination routes of microbial pathogens to fresh produce during field cultivation: A review. Food Microbiol..

[5] Uçar A., Yilmaz M.V., Çakiroglu F.P. (2016). Food Safety–Problems and Solutions.

[6] McFarland P., Checinska Sielaff A., Rasco B., Smith S. (2019). Efficacy of Food Safety Training in Commercial Food Service. J. Food Sci..

[7] Aladhadh M. (2023). A Review of Modern Methods for the Detection of Foodborne Pathogens. Microorganisms.

[8] Tomić A., Šovljanski O., Erceg T. (2023). Insight on Incorporation of Essential Oils as Antimicrobial Substances in Biopolymer-Based Active Packaging. Antibiotics.

[9] Almansour A.M., Alhadlaq M.A., Alzahrani K.O., Mukhtar L.E., Alharbi A.L., Alajel S.M. (2023). The Silent Threat: Antimicrobial-Resistant Pathogens in Food-Producing Animals and Their Impact on Public Health. Microorganisms.

[10] Chinemerem Nwobodo D., Ugwu M.C., Oliseloke Anie C., Al-Ouqaili M.T.S., Chinedu Ikem J., Victor Chigozie U., Saki M. (2022). Antibiotic resistance: The challenges and some emerging strategies for tackling a global menace. J. Clin. Lab. Anal..

[11] Tropea A. (2022). Microbial Contamination and Public Health: An Overview. Int. J. Environ. Res. Public Health.

[12] Lorenzo J.M., Munekata P.E., Dominguez R., Pateiro M., Saraiva J.A., Franco D. (2018). Main Groups of Microorganisms of Relevance for Food Safety and Stability: General Aspects and Overall Description. Innov. Technol. Food Preserv..

[13] Ferrari R.G., Rosario D.K.A., Cunha-Neto A., Mano S.B., Figueiredo E.E.S., Conte-Junior C.A. (2019). Worldwide Epidemiology of *Salmonella* Serovars in Animal-Based Foods: A Meta-analysis. Appl. Environ. Microbiol..

[14] Ehuwa O., Jaiswal A.K., Jaiswal S. (2021). *Salmonella*, Food Safety and Food Handling Practices. Foods.

[15] Willson N.L., Chousalkar K. (2023). Dominant *Salmonella* Serovars in Australian Broiler Breeder Flocks and Hatcheries: A Longitudinal Study. Appl. Environ. Microbiol..

[16] Heredia N., García S. (2018). Animals as sources of food-borne pathogens: A review. Anim. Nutr..

[17] Andino A., Hanning I. (2015). *Salmonella enterica*: Survival, colonization, and virulence differences among serovars. Sci. World J..

[18] Fàbrega A., Vila J. (2013). *Salmonella enterica* serovar Typhimurium skills to succeed in the host: Virulence and regulation. Clin. Microbiol. Rev..

[19] Hoelzer K., Moreno Switt A., Wiedmann M. (2011). Animal contact as a source of human non-typhoidal salmonellosis. Vet. Res..

[20] Back D.S., Shin G.W., Wendt M., Heo G.J. (2016). Prevalence of *Salmonella* spp. in pet turtles and their environment. Lab. Anim. Res..

[21] Cox N.A., Cason J.A., Richardson L.J. (2011). Minimization of *Salmonella* Contamination on Raw Poultry. Annu. Rev. Food Sci. Technol..

[22] Bacon R.T., Sofos J.N., Belk K.E., Hyatt D.R., Smith G.C. (2002). Prevalence and antibiotic susceptibility of *Salmonella* isolated from beef animal hides and carcasses. J. Food Prot..

[23] Zeng H., De Reu K., Gabriël S., Mattheus W., De Zutter L., Rasschaert G. (2021). *Salmonella* prevalence and persistence in industrialized poultry slaughterhouses. Poult. Sci..

[24] Reiter M.G.R., Fiorese M.L., Moretto G., López M.C., Jordano R. (2007). Prevalence of *Salmonella* in a poultry slaughterhouse. J. Food Prot..

[25] Dias M.R., Cavicchioli V.Q., Camargo A.C., Lanna F.G.P.A., Pinto P.S.D.A., Bersot L.D.S., Nero L.A. (2016). Molecular tracking of *Salmonella* spp. in chicken meat chain: From slaughterhouse reception to end cuts. J. Food Sci. Technol..

[26] Botteldoorn N., Heyndrickx M., Rijpens N., Grijspeerdt K., Herman L. (2003). *Salmonella* on pig carcasses: Positive pigs and cross contamination in the slaughterhouse. J. Appl. Microbiol..

[27] Olsen J.E., Brown D.J., Madsen M., Bisgaard M. (2003). Cross-contamination with *Salmonella* on a broiler slaughterhouse line demonstrated by use of epidemiological markers. J. Appl. Microbiol..

[28] Reta G.G., Lopes S.M., de Aquino N.S.M., Tondo E.C. (2023). Quantification of *Salmonella* transfer in cross-contamination scenarios found in chicken slaughterhouses. Food Microbiol..

[29] Bhandari R., Singh A.K., Bhatt P.R., Timalsina A., Bhandari R., Thapa P., Baral J., Adhikari S., Poudel P., Chiluwal S. (2022). Factors associated with meat hygiene-practices among meat-handlers in Metropolitan City of Kathmandu, Nepal. PLoS Glob Public Health.

[30] Montoro-Dasi L., Lorenzo-Rebenaque L., Marco-Fuertes A., Vega S., Marin C. (2023). Holistic Strategies to Control *Salmonella* Infantis: An Emerging Challenge in the European Broiler Sector. Microorganisms.

[31] Liu S., Tang J., Tadapaneni R.K., Yang R., Zhu M.J. (2018). Exponentially Increased Thermal Resistance of *Salmonella* spp. and *Enterococcus faecium* at Reduced Water Activity. Appl. Environ. Microbiol..

[32] Finn S., Condell O., McClure P., Amézquita A., Fanning S. (2013). Mechanisms of survival, responses and sources of *Salmonella* in low-moisture environments. Front. Microbiol..

[33] Kumar S., Singh M., Cosby D.E., Cox N.A., Thippareddi H. (2020). Efficacy of peroxy acetic acid in reducing *Salmonella* and *Campylobacter* spp. populations on chicken breast fillets. Poult. Sci..

[34] de Rezende H.C., de Lima M., Santos L.D. (2023). Peracetic acid application as an antimicrobial and its residual (HEDP): A holistic approach on the technological characteristics of chicken meat. Poult. Sci..

[35] Kitis M. (2004). Disinfection of wastewater with peracetic acid: A review. Environ. Int..

[36] Huertas J.P., Aznar A., Esnoz A., Fernández P.S., Iguaz A., Periago P.M., Palop A. (2016). High Heating Rates Affect Greatly the Inactivation Rate of *Escherichia coli*. Front. Microbiol..

[37] Rui L., Xiaoxi K., Lihui Z., Shaojin W. (2018). Inactivation kinetics of food-borne pathogens subjected to thermal treatments: A review. Int. J. Hyperth..

[38] Holah J.T., Lelieveld H.L.M., Mostert M.A., Holah J., White B. (2013). Cleaning and disinfection. Hygiene in Food Processing.

[39] Meyer B., Morin V.N., Rödger H.-J., Holah J., Bird C. (2010). Do European Standard Disinfectant tests truly simulate in-use microbial and organic soiling conditions on food preparation surfaces?. J. Appl. Microbiol..

[40] Lourenço C., Macdonald T.J., Gavriilidis A., Allan E., MacRobert A.J., Parkin I.P. (2018). Effects of bovine serum albumin on light activated antimicrobial surfaces. RSC Adv..

[41] Mastusaka Y., Kawabata J. (2010). Evaluation of Antioxidant Capacity of Non-Edible Parts of Some Selected Tropical Fruits. Food Sci. Technol. Res..

[42] Moore G., Blair I.S., McDowell D.A. (2007). Recovery and Transfer of *Salmonella Typhimurium* from Four Different Domestic Food Contact Surfaces. J. Food Prot..

[43] Tomić A., Šovljanski O., Nikolić V., Pezo L., Aćimović M., Cvetković M., Stanojev J., Kuzmanović N., Markov S. (2023). Screening of Antifungal Activity of Essential Oils in Controlling Biocontamination of Historical Papers in Archives. Antibiotics.

[44] Šovljanski O., Pezo L., Tomić A., Ranitović A., Cvetković D., Markov S. (2022). Formation of Predictive-Based Models for Monitoring the Microbiological Quality of Beef Meat Processed for Fast-Food Restaurants. Int. J. Environ. Res. Public Health.

[45] Aćimović M., Šovljanski O., Šeregelj V., Pezo L., Zheljazkov V.D., Ljujić J., Tomić A., Ćetković G., Čanadanović-Brunet J., Miljković A. (2022). Chemical Composition, Antioxidant, and Antimicrobial Activity of *Dracocephalum moldavica* L. Essential Oil and Hydrolate. Plants.

[46] Šovljanski O., Pezo L., Stanojev J., Bajac B., Kovač S., Tóth E., Ristić I., Tomić A., Ranitović A., Cvetković D. (2021). Comprehensive Profiling of Microbiologically Induced CaCO_3_ Precipitation by Ureolytic *Bacillus* Isolates from Alkaline Soils. Microorganisms.

[47] Mazzola P.G., Penna T.C., Martins A.M. (2003). Determination of decimal reduction time (D value) of chemical agents used in hospitals for disinfection purposes. BMC Infect Dis..

[48] Cebrián G., Condón S., Mañas P. (2017). Physiology of the Inactivation of Vegetative Bacteria by Thermal Treatments: Mode of Action, Influence of Environmental Factors and Inactivation Kinetics. Foods.

[49] Dash K.K., Fayaz U., Dar A.H., Shams R., Manzoor S., Sundarsingh A., Deka P., Khan S.A. (2022). A comprehensive review on heat treatments and related impact on the quality and microbial safety of milk and milk-based products. Food Chem. Adv..

[50] Hassan H., Iskandar C.F., Hamzeh R., Malek N.J., Khoury A.E., Abiad M.G. (2022). Heat resistance of *Staphylococcus aureus*, *Salmonella* sp., and *Escherichia coli* isolated from frequently consumed foods in the Lebanese market. Int. J. Food Prop..

[51] James C., Dixon R., Talbot L., James S.J., Williams N., Onarinde B.A. (2021). Assessing the Impact of Heat Treatment of Food on Antimicrobial Resistance Genes and Their Potential Uptake by Other Bacteria-A Critical Review. Antibiotics.

[52] Amado I.R., Vázquez J.A., Guerra N.P., Pastrana L. (2014). Thermal resistance of *Salmonella enterica*, *Escherichia coli* and *Staphylococcus aureus* isolated from vegetable feed ingredients. J. Sci. Food Agric..

[53] Stopforth J.D., Suhalim R., Kottapalli B., Hill W.E., Samadpour M. (2008). Thermal Inactivation D- and z-Values of Multidrug-Resistant and Non–Multidrug-Resistant *Salmonella* Serotypes and Survival in Ground Beef Exposed to Consumer-Style Cooking. J. Food Prot..

[54] Rajkowski K.T. (2012). Thermal inactivation of *Escherichia coli* O157:H7 and *Salmonella* on catfish and tilapia. Food Microbiol..

[55] Aryal M., Muriana P.M. (2019). Efficacy of Commercial Sanitizers Used in Food Processing Facilities for Inactivation of *Listeria Monocytogenes*, *E. Coli* O157:H7, and *Salmonella* Biofilms. Foods.

[56] Nagel G.M., Bauermeister L.J., Bratcher C.L., Singh M., McKee S.R. (2013). *Salmonella* and *Campylobacter* reduction and quality characteristics of poultry carcasses treated with various antimicrobials in a post-chill immersion tank. Int. J. Food Microbiol..

[57] Costa S.A.d.S., Paula O.F.P., de Silva C.R.G., Leao M.V.P., dos Santos S.S.F. (2015). Stability of antimicrobial activity of peracetic acid solutions used in the final disinfection process. Braz. Oral Res..

[58] Suo B., Shi C., Shi X. (2012). Inactivation and occurrence of sublethal injury of *Salmonella Typhimurium* under mild heat stress in broth. J. Für Verbraucherschutz Und Leb..

[59] Elpers L., Deiwick J., Hensel M. (2022). Effect of Environmental Temperatures on Proteome Composition of *Salmonella enterica* Serovar Typhimurium. Mol Cell Proteom..

[60] Abee T. (1999). Microbial stress response in minimal processing. Int. J. Food Microbiol..

